# Medical Education

**Published:** 1879-10

**Authors:** 


### EDITORIAL.

---

### MEDICAL EDUCATION.

At this season of the year the various Medical Colleges of our country scatter broadcast their annual crop of announcements as regularly as the trees shed their leaves. But while they attract the student with seductive statements of superior advantages, the older practitioner is apt to regard their allurements with a feeling of distrust. A circular of this sort too often furnishes the text over which he moralizes, concerning the American system of medical education, and perhaps he is inclined to join in the fashionable outcry concerning it.

There are certain disagreeable facts which stare him in the face, and which can not be blinked at nor disregarded. Not only are medical schools increasing rapidly, but they are already more numerous, and graduate more men in a shorter time than in any other country on the globe. In 1876 we had sixty-four schools to supply doctors to a little over forty million people. The United Kingdom of Great Britain has forty-one schools to about thirty million inhabitants. Germany has only twenty for forty-one millions, and in France the proportion is smaller still.

A further examination of statistics shows that we have now, on an average, one doctor for every 600 people, while

Canada has one to 1,200, Great Britain one to 1,672, France one to 1,814, and Germany one to about every 3,000 inhabitants. Moreover, foreign doctors are made much more deliberately than with us.

The Canadian or Englishman spends four years in study, besides starting with a better preparation. In continental countries it requires five years to complete the course, and occasionally six are demanded, while Young America skips through in half that time. Facts like these are suggestive, but by no means flattering, to our professional pride. Especially has the feeling found expression in the medical press, until now the topic is made almost conspicuous by its absence from the pages of any of our leading journals.

It behooves us, therefore, to notice these apparent or real defects in our present system, and face fairly the questions which present themselves. Without doubt, colleges have been rapidly multiplied, but this was partly necessitated by the extraordinary growth of the country. It should not be forgotten that the population has increased from about seven millions in 1810 to over forty millions in 1876, and instead of four schools, as then, we need of course many more at present.

It is argued that the number of physicians yearly turned out is too large, for the reason that we have already more to the population than can be found elsewhere. The comparison is not entirely just, however, for we have a far larger extent of territory over which medical men must be distributed. In 1870 there were with us only 9.8 persons to the square mile, while in the United Kingdom there were 249.9, in France 181.2, and in Germany 190. Comparing this, with statements made before, it appears that the number of physicians must, to a certain extent, be determined by the density of population. And this is not surprising. Where each man has a wide circuit, where the country is new, the roads often poor, and patients separated by long distances, it is but natural that the number should be larger than in thickly settled districts.

There are, however, certain important features of our system which admit of considerable improvement. A certain amount of preliminary training should be demanded, before professional study is undertaken. According to Professor Huxley, "It may be safely said that, with a large proportion of medical students, much of the first session is wasted in learning how to learn." And when the time comes to select a school, the novice frequently gives an undue preference to metropolitan institutions. He has a false notion that a large clinical experience is necessary from the first. The choice is frequently determined more by the size of the building pictured on the cover of the announcement than by any merit of the school itself. But in reality students have no business at a hospital, and in some countries are not admitted to them until after anatomy, physiology, chemistry, and materia medica have been disposed of. While at work upon these, a school which offers little or no clinical advantages is perhaps the best.

The fact should not be lost sight of, however, that when the American does study, he goes at it with all his might. He does that and nothing else. On the other hand, an Englishman mingles study with rowing, riding and the excitement of the Derby. The German takes his medicine deliberately, and digests each dose of lectures only after steeping it in the plentiful beer and tobacco smoke of the *Gasthaus*, where his corps meet every night. The Frenchman is even less diligent. Evening study is something foreign to his notions, and every one who has frequented the Cafe D'Harcourt or Jardin Bullier will appreciate how many hours in his life are worse than wasted.

But in spite of the difference in national characteristics, there is no disguising the fact that a course of three years is far too short, and some concerted effort should be made, not only to lengthen it, but to have the branches of the various curricula more systematically arranged.

Finally, the examination should be conducted by those who have no personal interest in the pupil or teacher. The Medical

Society of this State expressed the popular conviction when, in 1839, it resolved, "That the right of teaching ought to be separated as much as possible from the power of conferring degrees or license." When this is done with us, as it is in every other country, the various schools will vie with each other in honorable competition, and the worst features of our system will spontaneously vanish.

Out of this resolution ultimately grew the association of medical colleges, which by combined effort strives to meet the demands for a more perfect type of professional excellence. We are also gratified to recognize the efforts of the Buffalo Medical College in the direction of an improvement of the standard of education, by increasing the facilities for the acquisition of both a theoretical and practical knowledge of medical science. Every such attempt meets with earnest approbation from the leading physicians of the day, and shows that our system of medical training, though still imperfect, is being gradually improved to keep pace with the intellectual growth of the country.

